# Maximum Likelihood Estimation for Shape-restricted Single-index Hazard Models

**DOI:** 10.6339/22-jds1061

**Published:** 2022-11-04

**Authors:** Jing Qin, Yifei Sun, Ao Yuan, Chiung-Yu Huang

**Affiliations:** 1 Biostatistics Research Branch, National Institute of Allergy and Infectious Diseases, Maryland, U.S.A.; 2 Department of Biostatistics, Mailman School of Public Health, Columbia University, New York, U.S.A.; 3 Department of Biostatistics, Bioinformatics & Biomathematics, Georgetown University, Washington D.C., U.S.A.; 4 Department of Epidemiology & Biostatistics, University of California San Francisco, California, U.S.A.

**Keywords:** isotonic regression, pool-adjacent-violators algorithm, profile likelihood, semiparametric estimation

## Abstract

Single-index models are becoming increasingly popular in many scientific applications as they offer the advantages of flexibility in regression modeling as well as interpretable covariate effects. In the context of survival analysis, the single-index hazards models are natural extensions of the Cox proportional hazards models. In this paper, we propose a novel estimation procedure for single-index hazard models under a monotone constraint of the index. We apply the profile likelihood method to obtain the semiparametric maximum likelihood estimator, where the novelty of the estimation procedure lies in estimating the unknown monotone link function by embedding the problem in isotonic regression with exponentially distributed random variables. The consistency of the proposed semiparametric maximum likelihood estimator is established under suitable regularity conditions. Numerical simulations are conducted to examine the finite-sample performance of the proposed method. An analysis of breast cancer data is presented for illustration.

## 1 Introduction

Single-index models have received much attention in many fields, including medicine, economics, finance, and environmental science. The single-index models can be viewed as a natural extension of the generalized linear models, where the link function is left unspecified and the covariate effects are summarized using a one-dimensional variable, referred to as the index (Powell et al., 1989; Härdle and Stoker, 1989; Ichimura, 1993; Härdle et al., 1993). In this paper, we consider single-index models for right-censored survival data. Let T denote the failure time of interest and let X be a p×1 vector of covariates. The popular Cox proportional hazards model assumes that, given X=x, the conditional hazard function λ(t∣x) satisfies

$$\lambda (t\;|\;x)=\lambda (t)\;\text{exp}(x^{T}\beta ),$$

where β is a *p*-dimensional vector of regression parameters and the baseline hazard λ(t) is left unspecified. The Cox model imposes an exponential functional form for the covariate effects on the hazard of failure, so that the covariates have a linear effect on the log hazard function. As pointed out by many authors, including Prentice (Prentice and Self, 1983), such assumption can be easily violated and it is desirable to consider a more flexible class of regression models

(1) $$\lambda (t\;|\;x)=\lambda (t)\;\text{exp}\{\phi (x^{T}\beta )\},$$

where ϕ is the link function. This model allows characterization of covariate effects on the risk of experiencing the failure event in a parsimonious way via a single index $x^{T}\beta$. When ϕ is known, the partial likelihood method (Cox, 1975) can be applied directly to estimate the regression parameters β with right-censored survival data.

When the link function ϕ is unspecified, local partial likelihood methods, derived by employing either spline or local polynomial smoothing approximations for the unknown link function, have been proposed for estimating the link function in the case where X is an univariate continuous variable (Tibshirani and Hastie, 1987; Fan et al., 1997; Chen and Zhou, 2007). The local partial likelihood method can be inefficient because only data from individuals with covariate values in the neighborhood of $x_{0}$ are used to estimate $\phi (x_{0})$. Gentleman and Crowley (Gentleman and Crowley, 1991) considered the local version of the full likelihood and developed an estimation procedure that alternates between estimating λ and estimating ϕ. Lately, Chen et al (Chen et al., 2010) proposed a global partial likelihood method that use all observations to estimate the value of the link function at any $x_{0}$. Statistical methods for Model (1) with multi-dimensional X have been developed in the same spirit as that for the univariate case. In particular, Wang et al (Wang et al., 2009) described an algorithm that iterates between maximizing the local partial likelihood function with respect to the smoothed approximation of ϕ for a given β and maximizing the global partial likelihood with respect to β with the estimated ϕ. Huang and Liu (Huang and Liu, 2006), on the other hand, proposed to approximate ϕ with cubic splines, thus reduces to a parametric model for ϕ which can be estimated directly using standard partial likelihood methods. However, large sample properties of the spline based approach are not well studied as, in theory, an infinite number of spline bases may be needed to span the unknown link function.

In many applications, it is desirable to impose shape restrictions, such as monotonicity and concavity, on the form of the covariate effects. Incorporating such a constraint can lead to improved efficiency and reduction in model complexity while allowing for more straightforward interpretation. For example, in dose finding trials for combination therapies, the marginal dose-response curve is often believed to be monotonic. While estimation procedures have been proposed for estimating the usual single-index models under shape-constraints with complete data (Foster et al., 2013; Groeneboom and Hendrickx, 2019), less attention has been paid to single-index hazards models under shape-constraints with right-censored data. Recently, Chung et al (Chung et al., 2018) considered a Cox model with shape constraints on the covariate effects, where, conditioning on covariates X=x and Z=z, the hazard function is assumed to take the form

λ(t∣x,z)=λ(t)exp{ψ(x)+zβ},

with ψ(x) being an unspecified monotone function of the univariate variable X. The authors modified the iterative convex minorant algorithm of Jongbloed (Jongbloed, 1998) and proposed a pseudo-iterative convex minorant algorithm to maximize the partial likelihood. Specifically, the partial likelihood is sequentially approximated by quadratic functions and, as a result, the pool adjacent violators algorithm (PAVA) (Ayer et al., 1955) can be readily applied. In this paper, we fill in the gap by studying the single index model (1) with an unspecified, monotonic link function ϕ. Instead of maximizing the partial likelihood, we embed the full likelihood function in the isotonic regression problem with exponentially distributed random variables and develop an iterative convex maximization algorithm. Our method provides a computationally stable estimation and, as demonstrated by the simulation studies, offers substantial efficiency gains over the Cox proportional hazards model when the link function is misspecified.

## 2 An Iterative Convex Maximization Algorithm

For ease of discussion, we write r(z)=exp{−ϕ(z)} and assume that r(z) is an unspecified non-decreasing function; or, equivalently, ϕ(z) is non-increasing in z. We adopt the convention with upper case letters, such as (Y, Δ, X), for random variables, and lower case letters, such as (y, δ, x) for observed values of (Y, Δ, X). With a minor modification, the estimation procedure discussed below can be applied to deal with the case where ϕ is non-decreasing or unimodal. Under the single-index model (1), the conditional hazard function of the survival time T is given by

$$\lambda (t\;|\;x)=\lambda (t)∕r(x^{T}\beta ),\;\;t\in [0,\tau ],$$

where one is interested in making inference about the survival time distribution on a prespecified time interval [0,τ]. Define the baseline cumulative hazard function $\Lambda (t)=\int_{0}^{t}\lambda (u)du$. It is easy to see that, for any constant k>0, the pair $r^{*}(z)=kr(z)$ and $\lambda^{*}(t)=k\lambda (t)$ gives the same model as r(z) and λ(t), owing to the semiparametric nature of the Cox model. Moreover, the pair $r^{*}(z)=r(kz)$ and $\beta^{*}=k^{-1}\beta$ also yields the same model as that from r(z) and β. In this paper, we impose Λ(τ)=1 and ‖β‖=1 to ensure model identifiability and note that the hazard function for the reference group (X=0) is given by Λ(t)∕r(0). A proof of the model identifiability is given in the Appendix.

In practice, the observation of the survival time T is usually subject to right censoring due to study end or premature dropout. Thus, instead of observing the actual value of T, we observe the possibly censored survival time Y=min(T,C), where C is the time of censoring. In many applications, it is reasonable to assume that C is independent of T given the observed covariates X. Denote by Δ=I(T≤C) the indicator function of a failure event. Assume that the observed data {$(y_{i},\delta_{i},x_{i}),i=1,\ldots ,n$} are independent realizations of (Y, Δ, X). Then the likelihood function based on the observed data is

$$𝓛(\beta ,r,\Lambda )=\prod_{i=1}^{n}{\left\{\frac{\lambda (y_{i})}{r(x_{i}^{T}\;\beta )}\right\}}^{\delta_{i}}\;\text{exp}\left\{-\frac{\Lambda (y_{i})}{r(x_{i}^{T}\;\beta )}\right\}.$$

Given β and r(⋅), the full likelihood 𝓛 is maximized by the Breslow-type estimator

$${\hat{\Lambda }}^{*}(t)=\sum_{i=1}^{n}\frac{\delta_{i}I(y_{i}\leq t)}{\sum_{j=1}^{n}I(y_{j}\geq y_{i})∕r(x_{j}^{T}\beta )}.$$

It is easy to see that replacing Λ with ${\hat{\Lambda }}^{*}$ in the full likelihood 𝓛 yields the partial likelihood

$$\prod_{i=1}^{n}{\left\{\frac{1∕r(x_{i}^{T}\beta )}{\sum_{j=1}^{n}I(y_{j}\geq y_{i})∕r(x_{j}^{T}\beta )}\right\}}^{\delta_{i}}.$$

However, direct maximization of the partial likelihood under the monotonicity constraint of r(⋅) is challenging and the conventional isotonic regression methods are not directly applicable.

In what follows, we consider semiparametric maximum likelihood estimation for Model (1) under the monotonicity constraint of r(⋅). We note that the maximum likelihood estimator derived without imposing the monotonicity constraint on the link function can be inconsistent. It is known that the nonparametric maximum likelihood estimate of a function concentrates its masses only on (some or all of) the observed data points. Without constraints, the masses may take any values and make the likelihood arbitrarily large. Thus we impose the monotone constraint on the link function r(⋅). Instead of directly maximizing the partial likelihood, we embed the full likelihood into the isotonic regression problems with exponential random variables and apply the pool adjacent violators algorithm (PAVA) to obtain the constrained maximizer for the link function. The proposed algorithm uses an inner loop to calculate {Λ(⋅),r(⋅)} that maximize the full likelihood function 𝓛(β,r,Λ) at a given value of β and an outer loop to maximize the profile likelihood with respect to β.

Specifically, given β and r(⋅), we derive the maiximiser ${\hat{\Lambda }}^{*}$ of the full likelihood and estimate Λ by $\hat{\Lambda }(t)={\hat{\Lambda }}^{*}(t)∕{\hat{\Lambda }}^{*}(\tau )$, where rescaling is carried out to ensure the identifiability condition Λ(τ)=1. Next, for given β and Λ(⋅), we define $Z_{i}\;=\;X_{i}^{T}\beta$ and sort the observed data {$(y_{i},\delta_{i},x_{i}),i=1,\ldots ,n$} according to the value of $z_{i}$’s, so that $x_{1}^{T}\beta \leq \cdots \leq x_{n}^{T}\beta$. Write $\eta_{i}=\Lambda (y_{i})$ and $r_{i}=\text{exp}\{-\phi (z_{i})\}=\text{exp}\{-\phi (x_{i}^{T}\beta )\}$. Then, for fixed β and Λ, the full likelihood is proportional to

$${𝓛}_{r}=\prod_{i=1}^{n}{\left(\frac{1}{r_{i}}\right)}^{\delta_{i}}\;\text{exp}\left(-\frac{\eta_{i}}{r_{i}}\right)$$

We maximize ${𝓛}_{r}$ with respect to $r_{i}$’s under the monotone constraint $r_{1}\;\leq \;r_{2}\leq \cdots \;\leq \;r_{n}$ by embedding into the isotonic regression problem with exponential random variables. Let $z_{1}^{*},\ldots ,z_{L}^{*}$ be the ordered, distinct values of the $z_{i}$’s from *uncensored* observations. We further define the subintervals $I_{1}=(-\infty ,z_{1}^{*}],\ldots ,I_{L}=(z_{L-1}^{*},z_{L}^{*}]$, and $I_{L+1}=(z_{L}^{*},\infty )$. It’s easy to see that, if the event time of the *i*th subject is censored, the contribution of the observation to the likelihood is given by exp($\text{exp}(-\eta_{i}∕r_{i})$), so that the likelihood increases with $r_{i}$. If $z_{i}$ falls into the subinterval $I_{l}$, then, under the monotone constraint, maximization is achieved by setting $r_{i}\;=\;r(z_{l}^{*})$. In other words, ${𝓛}_{r}$ is maximized by setting $r_{i}=r_{l}$ if $z_{i}\in I_{l}$, l≤L, and $r_{i}=\infty$ if $z_{i}>z_{L}^{*}$. To perform isotonic regression, we exclude data from individuals whose z value is greater than $z_{L}^{*}$ and consider the following likelihood based on the reduced dataset

$${𝓛}_{r}^{*}=\prod_{l=1}^{L}{\left(\frac{1}{r_{l}^{*}}\right)}^{m_{l}^{d}}\;\text{exp}\left(-\frac{m_{l}^{d}{\bar{\eta }}_{l}}{r_{l}^{*}}\right),$$

where $r_{l}^{*}$ is the value of r(z) evaluated at $z=z_{l}^{*}$, $m_{l}^{d}=\sum_{i=1}^{n}\delta_{i}I(z_{i}=z_{l}^{*})$ is the number of uncensored individuals whose Z value is $z_{l}^{*}$, and ${\bar{\eta }}_{l}=\frac{1}{m_{l}^{d}}\sum_{i=1}^{n}\Lambda (y_{i})I(z_{i}\in I_{l})$, l=1,…,L. Note that the numerator of ${\bar{\eta }}_{l}$ include data from all individual, either censored or uncensored, but the denominator only include data from uncensored individuals. Maximisation of ${𝓛}_{r}^{*}$ subject to the monotone constraint can be viewed as an isotonic regression problem because ${𝓛}_{r}^{*}$ is mathematically equivalent to the likelihood of a sequence of L independent trials, where the outcomes are exponentially distributed with means satisfying $r_{1}^{*}\leq r_{2}^{*}\leq \cdots \leq r_{L}^{*}$ and the sample size of the lth trial is $m_{l}^{d}$. As pointed out in Chapter 1 of Robertson et al (Robertson et al., 1988), the pool-adjacent-violators algorithm (PAVA) (Ayer et al., 1955) can be used to solve exponential family isotonic regression problems. Hence, given β and Λ(⋅), we propose to maximize ${𝓛}_{r}^{*}$ with the PAVA estimator,


$${\hat{r}}_{l}^{*}=\max_{k\leq l}\underset{l\leq q}{\text{min}}\frac{\sum_{k\leq l\leq q}w_{l}{\bar{\eta }}_{l}}{\sum_{k\leq l\leq q}w_{l}}.$$

Thus we obtain $\hat{r}(z_{i})={\hat{r}}_{l}^{*}$ for $z_{i}\in I_{l}$, l≤L, and $\hat{r}(z_{i})={\hat{r}}_{L}^{*}$ for $z_{i}\in I_{L+1}$.

For a given β, the estimation algorithm for {Λ(⋅),r(⋅)} is summarized below. We alternate between (M1) and (M2) below repeatedly until some convergence criteria are met. Specifically, suppose the value of parameters in the *b*th step is ($\Lambda^{\left(b\right)}$, $r^{\left(b\right)}$). Then in the (b+1)th step, **(M1)** Calculate ${\bar{\eta }}_{l}^{\left(b+1\right)}=\frac{1}{m_{l}^{d}}\sum_{i=1}^{n}\Lambda^{\left(b\right)}(y_{i})I(z_{i}\in I_{l})$. Apply PAVA to obtain

$$r_{l}^{\left(b+1\right)}=\max_{k\leq l}\underset{l\leq q}{\text{min}}\frac{\sum_{k\leq l\leq q}m_{l}^{d}\;{\bar{\eta }}_{l}^{\left(b+1\right)}}{\sum_{k\leq l\leq q}m_{l}^{d}}.$$

Set $r^{\left(b+1\right)}(z_{i})=r_{l}^{\left(b+1\right)}$ for $z_{i}\in I_{l}$, l≤L, and $r^{\left(b+1\right)}(z_{i})=r_{L}^{\left(b+1\right)}$ for $z_{i}\in I_{L+1}$. **(M2)** Update Λ with the Breslow-Type estimator

$$\Lambda^{(b+1)*}(t)=\sum_{i=1}^{n}\frac{\delta_{i}I(y_{i}\leq t)}{\sum_{j=1}^{n}{I(y_{j}\geq y_{i})∕r}^{\left(b+1\right)}(z_{j})}$$

to obtain $\Lambda^{\left(b+1\right)}(t)=\Lambda^{(b+1)*}(t)∕\Lambda^{(b+1)*}(\tau )$.

For fixed β, we iterate between (M1) and (M2) until convergence. Denote the limit by ($\hat{\Lambda }(\cdot ;\beta ),\hat{r}(\cdot ;\beta )$).

Finally, we plug in $\hat{\Lambda }(\cdot ;\beta )$ and $\hat{r}(\cdot ;\beta )$ back to the full likelihood function 𝓛(β,r,Λ) to obtain the profile likelihood function ${𝓛}_{p}(\beta )=𝓛(\beta ,\hat{r}(\cdot ;\beta ),\hat{\Lambda }(\cdot ;\beta ))$. For a given β, $\hat{r}(\cdot ;\beta )$ is only uniquely defined at the ordered, distinct values of $x_{i}^{T}\beta$ from uncensored observations. With a finite sample, the maximizer of ${𝓛}_{p}(\beta )$ is not unique, as ${𝓛}_{p}(\beta )$ only depends on the ordering of $x_{i}^{T}\beta$ from uncensored observations induced by β. As shown in Theorem 1 below, the maximizer converges to the true parameters $\beta_{0}$ as the sample size goes to infinity. To account for the constraint ‖β‖=1, we use the spherical coordinate system to represent β on the unit sphere $𝓑=\{\beta \;:\;\|\beta \|=1,\beta \in R^{p}\}$. Following Balabdaoui et al (Balabdaoui et al., 2019), we use the following map to reduce the parameters to a (p−1)-dimensional vector, $S,:{\left[0,\pi \right]}^{\left(p-2\right)}\times [0,2\pi ]\mapsto 𝓑$; θ↦β, where $\theta =(\theta_{1},\theta_{2},\ldots ,\theta_{p-1})$, and

$$\beta \;=\;(\cos(\theta_{1}),\;\sin(\theta_{1})\;\cos(\theta_{2}),\ldots ,\sin(\theta_{1})\cdots \sin(\theta_{p-2})\;\cos(\theta_{p-1}),\;\;\sin(\theta_{1})\cdots \sin(\theta_{p-2})\;\sin(\theta_{p-1})).$$

Maximization of ${𝓛}_{p}(\beta )$ can be implemented using Nelder-Mead’s downhill simplex method (Nelder and Mead, 1965) with respect to ($\theta_{1},\theta_{2},\ldots ,\theta_{p-1}$). Different initial values can be used in the optimization for improved performance.

Let 𝓡 be the collection of monotone increasing functions on R, and 𝓐 be a collection of monotone increasing functions on $R^{+}$ such that the function takes value 1 at τ. Denote by ($\hat{\beta }$, $\hat{r}$, $\hat{\Lambda }$) the maximum likelihood estimator of the true parameters ($\beta_{0}$, $r_{0}$, $\Lambda_{0}$), that is,

$$(\hat{\beta },\;\hat{r},\;\hat{\Lambda })={\text{arg max}}_{\left(\beta ,r,\Lambda )\in (𝓑,𝓡,𝓐\right)}𝓛(\beta ,\;r,\;\Lambda ).$$

The consistency of the maximum likelihood estimator ($\hat{\beta }$, $\hat{r}$, $\hat{\Lambda }$) is stated in Theorem 1, with proof given in the Appendix.

**Theorem 1.** *Let* [$z_{1}$, $z_{2}$] *be a bounded interval in the support of* $X^{T}\beta_{0}$. *Under conditions* (C1)∼(C4) *in the* Appendix, *as* n→∞, *we have*

$$\hat{\beta }\overset{a.s.}{\to }\beta_{0}\;\;,\;\;\underset{z\in [z_{1},z_{2}]}{\text{sup}}|\;\hat{r}(z)-r_{0}(z)\;|\overset{a.s.}{\to }0\;\;,\;\;\underset{t\in [0,\tau ]}{\text{sup}}|\;\hat{\Lambda }(t)-\Lambda_{0}(t)\;|\overset{a.s.}{\to }0.$$

For single index models where the nonparametric component is estimated by nonparametric maximum likelihood estimation under shape constraints, the $\sqrt{n}$-convergence rate and asymptotic normality of the estimator for regression parameters is an open question (Huang and Wellner, 1997; Murphy et al., 1999; Groeneboom and Hendrickx, 2018). Our estimation procedure encounters similar technical challenges. The main reason is that $\hat{r}$ is a step function which is not smooth, and the regression part is bundled inside it. As a result, the asymptotic distribution of ($\hat{\beta }$, $\hat{r}$, $\hat{\Lambda }$) requires further investigation. Let [$z_{1}$, $z_{2}$] be a bounded interval in the support of $X^{T}\beta_{0}$. Define $\|{\hat{r}-r}_{0}\|={\left[\int_{z_{1}}^{z_{2}}{\left\{\hat{r}(z)-r_{0}(z)\right\}}^{2}dz\right]}^{1∕2}$ and $\|{\hat{\Lambda }-\Lambda }_{0}\|={\left[\int_{0}^{\tau }{\left\{\hat{\Lambda }(t)-\Lambda_{0}(t)\right\}}^{2}dt\right]}^{1∕2}$. In Theorem 2, we show that the convergence rate of ($\hat{\beta }$, $\hat{r}$, $\hat{\Lambda }$) is at least $n^{1∕3}$. The proof of Theorem 2 is given in the Supplementary Material.

**Theorem 2.** *Under conditions* (C1)∼(C5) *in the* Appendix, *we have*

$$\|\hat{\beta }-\beta_{0}\|+\|\hat{r}-r_{0}\|\;+\;\|\hat{\Lambda }-\Lambda_{0}\|=O_{p}(n^{-1∕3}).$$

It is worthwhile to point out that when the covariates have elliptically symmetric distribution, fitting a Cox model to the data yields consistent estimate of the direction of $\beta_{0}$. The result is summarized in Proposition 1 and the proof is given in the Appendix.

**Proposition 1.** *Let* ${\hat{\beta }}_{P}$ *be the maximum partial likelihood estimator under the usual Cox model with a (potentially misspecified) exponential link function,* $\lambda (t\;|\;x)=\lambda (t)\;\text{exp}(x^{T}\beta )$. *If* X *has an elliptically symmetric distribution and the censoring is completely random, then* $\beta_{0}$ *can be consistently estimated by* $-{\hat{\beta }}_{P}∕\|{\hat{\beta }}_{P}\|$.

## 3 Simulation Studies

We conduct simulation studies to evaluate the performance of the proposed method. Given covariates X, we generated survival times from the Weibull distribution with shape parameter 2 and scale parameter $\sqrt{r(X^{T}\beta^{*})}$, where $r(X^{T}\beta^{*})=\text{exp}\{{|X^{T}\beta^{*}|}^{a}\text{sign}(X^{T}\beta^{*})\}$. The hazard function is $\lambda (t\;|\;X)=2t\;\text{exp}\{-{|X^{T}\beta^{*}|}^{a}\text{sign}(X^{T}\beta^{*})\}$. We included p covariates and set $\beta^{*}=(\beta_{1}^{*},\ldots ,\beta_{p}^{*})$, where $\beta_{2m-1}^{*}=-1$ and $\beta_{2m}^{*}=1$ for m≥1, thus the true value is $\beta_{0}=\beta^{*}∕\sqrt{p}$. We considered the following scenarios: (I) p=2, $X_{j}$ were generated from the exponential distribution with rate parameter 1, denoted by exp(1), for j=1,2. (II) p=2, $X_{j}$ were generated from the standard normal distribution N(0,1) for j=1,2. (III) p=5, $X_{1}$, $X_{2}$ were generated from exp(1), $X_{4}$, $X_{5}$ were generated from N(0,1), and $X_{3}$ was generated from the Bernoulli distribution with success probability 1/2. (IV) p=5, $X_{1},\ldots ,X_{5}$ were generated from N(0,1). We also considered three cases under each scenario, that is, (A) a=1∕5, (B) a=1∕3, and (C) a=1. The censoring time was set as $C=\text{min}(C^{*},\tau )$, where $C^{*}$ was generated from exponential distributions with rate parameters $\lambda_{C}$, and $\lambda_{C}$ and τ were chosen to yield approximately 25% and 50% censoring rates. In each simulation, we generated 1000 datasets with sample sizes of 200 and 800. We compared the proposed method with the negative normalized coefficients from maximum partial likelihood estimator (MPLE) assuming an exponential link function. In the Supplementary Material, we also included the simulation results of an estimator without imposing the monotone constraint. Specifically, the estimator replaces the PAVA estimator of the link function with a kernel smoothing estimator. More details can be found in the Supplementary Material.

The non-smoothness of the profile likelihood function precludes the use of methods that utilize derivative because its derivative does not exist. Thus we considered applying Nelder-Mead’s method, which only requires the value of likelihood functions. Moreover, we used multiple initial values to improve the search for the maximizer. The first initial value of the Nelder-Mead algorithm is chosen as the negative normalized MPLE transformed to a (p−1)-dimensional vector via the map $S^{-1}$. When the solution is obtained, we further add a small perturbation (e.g., a random vector whose elements are generated from the uniform distribution on [−0.5, 0.5]) to the solution. We then run the Nelder-Mead’s algorithm with the perturbed solution as the initial value. If the likelihood function value is larger than that of the previous step, we replace the estimated parameters with the solution from the current step. We repeat this procedure twenty times and use the parameter value that yields the largest profile likelihood. In our current implementation, we applied the R function optim (R Core Team, 2020) for the Nelder-Mead algorithm to maximize ${𝓛}_{p}(\beta )$. To obtain {r, Λ} that maximize 𝓛(β,r,Λ) at each value of β, we applied the R function squarem in the package SQUAREM (Du and Varadhan, 2020), which is used to accelerate the convergence of general fixed-point iterations. We used the stopping criteria from the default setting in each function.

The results are reported in Table 1 and 2. In Scenario I, the covariates were generated from the exponential distribution, and the survival data was not generated from the Cox model. It can be observed that, when the link function is misspecified (i.e., a=1∕3 and a=1∕5, MPLE has substantially larger bias and variance compared to the proposed approach, and the bias does not decrease as the sample size increases; when the link function is correctly specified, the MPLE has smaller variances. In Scenarios II and IV, the covariates were generated from the normal distribution. Both methods yield small biases, and the variance decreases as the sample size increases. This is consistent with Proposition 1, that is, when the covariates have elliptically symmetric distribution, the negative normalized MPLE is consistent for $\beta_{0}$ even if the proportional hazards model assumption is violated. When the link function is misspecified, the proposed method has smaller variances; when the link function is correctly specified, the MPLE has smaller variances. In Scenario III, we include more covariates generated from different types of distributions. The biases and variances of the proposed estimator decrease as the sample size increases. However, when the link function is misspecified, the biases of MPLE remain large when n=800, and the variances of MPLE are larger compared to the proposed method. In summary, the proposed method performs well and outperforms MPLE when the assumption on the link function does not hold.

## 4 Breast Cancer Data Example

The proposed method is applied to a multicenter randomized clinical trial conducted by the German Breast Cancer Study Group (Schumacher et al., 1994). The aim of the trial was to compare the time-to-event outcomes between different treatment modalities. The data used in this paper to illustrate our findings are available in the R package mfp on the Comprehensive R Archive Network (Ambler and Benner, 2015). The primary outcome is the recurrence-free survival time, which is a composite endpoint of breast cancer recurrence and death. The median follow-up was 56 months. During the study period, 299 of the 686 patients had disease recurrence or died. The covariates included in the model are hormonal treatment (yes/no), tumor size, tumor grade (1/2/3), and the number of positive lymph nodes. To ensure stable numerical performance, we standardize the covariates taking numeric values to have zero mean and unit variance.

The conventional method to analyze this data is the standard Cox regression, which uses a pre-specified monotonic link function. By using an unspecified monotonic link function, we allow greater flexibility than the standard Cox regression. Moreover, compared to models with non-monotonic link functions, the use of monotone link leads to an easier interpretation in practical applications. Table 3 reports the coefficients that are normalized to have a unit norm. We reported $-\hat{\beta }$ from the proposed method and ${\hat{\beta }}_{P}∕\|{\hat{\beta }}_{P}\|$ from the Cox model, and thus a positive coefficient indicates a larger value of the covariate is associated with a higher risk. To ensure identifiability, we assume the coefficient of hormonal therapy is negative. In both Cox model and monotone single index model, hormonal therapy is associated with a lower survival risk; larger tumors, higher tumor grades, and more positive lymph nodes are associated with higher risks. The Cox model estimates a much smaller effect for the positive lymph node count relative to other covariates.

**Figure 1:**
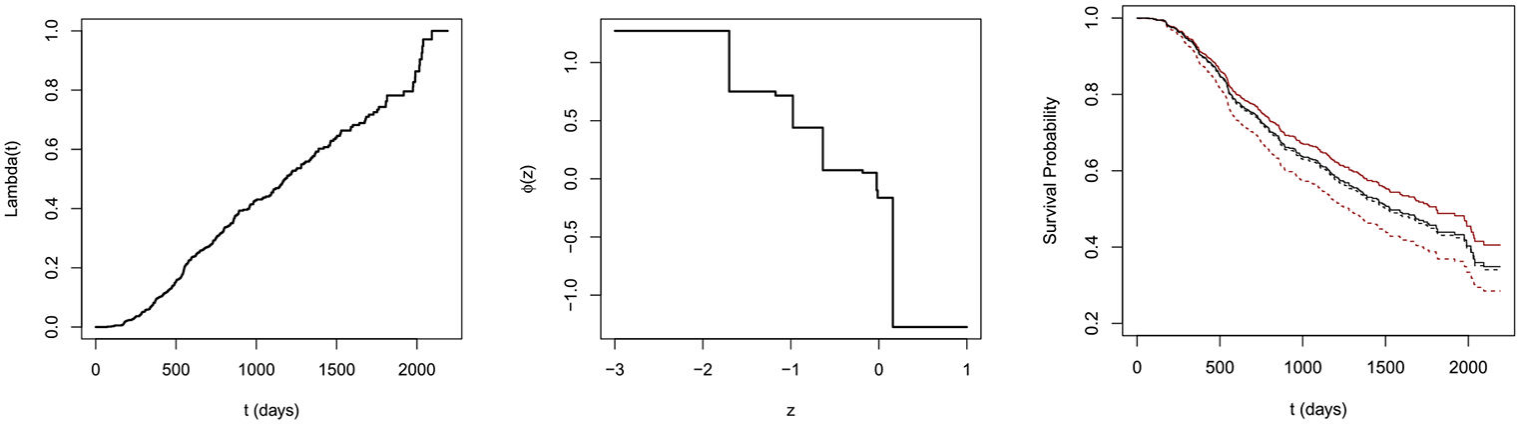
Estimated functions for the German Breast Cancer Study. The left and middle panel shows the estimated baseline cumulative hazard function Λ and link function ϕ, respectively. In the right panel, black lines are the predicted survival probabilities using the proposed model; the red lines are the predicted survival probabilities using the Cox model; the dashed lines are patients without hormonal therapy; the solid lines are patients with hormonal therapy.

The estimated hazard (normalized to have $\Lambda_{0}(\tau )=1$, τ=6 years) and estimated link function are reported in Figure 1. The shape of the estimated link function $\hat{\phi }(z)=-\log\;\hat{r}(z)$ provides evidence against the assumption of the exponential link function in the Cox model. In this case, the proposed method may yield less biased estimates. To provide more insight into the difference between the proposed method with the Cox model, we plot the predicted survival probability of two hypothetical patients in Figure 1. The two patients have tumors grade 3, and the other covariates are set to be the median values among grade 3 patients; one of them undergoes hormonal treatment while the other does not. The proposed method yields a smaller difference in survival curves compared to the Cox proportional hazards model. When predicting the survival probability, the proposed method is expected to be more robust than the Cox model under model misspecification. The difference in the predicted survival functions using the two methods may suggest a potential violation of the Cox model assumption.

## 5 Remarks

This paper focuses on semiparametric maximum likelihood estimation of the single-index hazards model under a shape-constraint. In our method, the link function is obtained from the PAVA algorithm and is not smooth. One may also consider the monotone splines (see, for example, Wang and Yan, 2021) to estimate the link function; this will be studied in our future work. Extensions of the proposed estimation procedure to the partially linear single-index hazards model under the same shape-constraint are straightforward, and their asymptotic properties will be studied elsewhere.

## Supplementary Material

supplemental materials

code

## Figures and Tables

**Table 1:** Summary of simulation studies (n=200).

		a = 1/5, cen = 25%				a = 1/3, cen = 25%				a = 1, cen = 25%			
		Proposed		MPLE		Proposed		MPLE		Proposed		MPLE	
		Bias	SE	Bias	SE	Bias	SE	Bias	SE	Bias	SE	Bias	SE
I	${\hat{\beta }}_{1}$	2	60	64	107	2	69	60	96	2	88	−1	51
	${\hat{\beta }}_{2}$	−3	61	45	93	−5	71	45	83	−9	89	−4	52
II	${\hat{\beta }}_{1}$	3	53	10	78	2	61	9	70	4	63	4	43
	${\hat{\beta }}_{2}$	−1	53	1	77	−3	62	2	69	−2	63	1	43
III	${\hat{\beta }}_{1}$	6	68	−5	92	4	71	−6	80	−3	52	−0.2	36
	${\hat{\beta }}_{2}$	−6	71	11	95	−5	73	10	82	−5	52	−0.1	37
	${\hat{\beta }}_{3}$	17	119	4	162	16	119	4	140	11	90	7	65
	${\hat{\beta }}_{4}$	−7	76	5	100	−10	77	10	88	−6	61	−4	43
	${\hat{\beta }}_{5}$	8	94	100	102	10	92	83	89	4	54	0.1	37
IV	${\hat{\beta }}_{1}$	−1	70	8	88	1	71	7	76	0.3	50	3	36
	${\hat{\beta }}_{2}$	−10	70	−14	87	−13	70	−11	76	−7	50	−4	36
	${\hat{\beta }}_{3}$	4	67	2	89	2	68	0.2	76	−1	49	−2	36
	${\hat{\beta }}_{4}$	−7	68	−12	88	−5	70	−8	75	−2	50	−1	35
	${\hat{\beta }}_{5}$	7	69	9	90	8	71	7	79	6	51	1	36
		a = 1/5, cen = 50%				a = 1/3, cen = 50%				a = 1, cen = 50%			
		Proposed		MPLE		Proposed		MPLE		Proposed		MPLE	
		Bias	SE	Bias	SE	Bias	SE	Bias	SE	Bias	SE	Bias	SE
I	${\hat{\beta }}_{1}$	3	84	95	131	5	97	85	118	5	107	0.1	64
	${\hat{\beta }}_{2}$	−7	87	65	108	−8	97	61	99	−11	111	−6	65
II	${\hat{\beta }}_{1}$	−0	75	3	91	4	83	3	82	3	77	−0.4	51
	${\hat{\beta }}_{2}$	−8	76	−8	90	−6	83	−7	82	−6	77	−4	52
III	${\hat{\beta }}_{1}$	−2	100	1	115	−1	98	1	102	1	63	3	45
	${\hat{\beta }}_{2}$	−13	100	−6	116	−13	96	−4	103	−5	64	−3	45
	${\hat{\beta }}_{3}$	25	152	11	187	27	153	8	162	8	106	3	73
	${\hat{\beta }}_{4}$	−15	107	10	124	−15	105	13	110	−12	75	−7	54
	${\hat{\beta }}_{5}$	30	127	108	122	22	120	88	107	6	60	−0.2	42
IV	${\hat{\beta }}_{1}$	5	94	17	107	4	93	12	94	0.3	60	3	42
	${\hat{\beta }}_{2}$	−9	90	−14	108	−8	91	−11	94	−6	62	−3	45
	${\hat{\beta }}_{3}$	4	95	8	108	6	91	6	94	0.2	62	1	44
	${\hat{\beta }}_{4}$	−19	91	−16	105	−19	88	−12	91	−7	58	−2	41
	${\hat{\beta }}_{5}$	11	93	9	105	9	90	7	93	8	63	2	43

Note: MPLE stands for the maximum partial likelihood estimator and cen stands for the censoring rate. Bias and SE are the empirical bias (×1000) and empirical standard deviation (×1000) of 1000 simulated datasets, respectively.

**Table 2:** Summary of simulation studies (n=800).

		a = 1/5, cen = 25%				a = 1/3, cen = 25%				a = 1, cen = 25%			
		Proposed		MPLE		Proposed		MPLE		Proposed		MPLE	
		Bias	SE	Bias	SE	Bias	SE	Bias	SE	Bias	SE	Bias	SE
I	${\hat{\beta }}_{1}$	0.1	19	77	61	1	26	74	55	1	44	2	24
	${\hat{\beta }}_{2}$	−0.4	19	65	50	−0.1	26	64	45	−2	44	1	24
II	${\hat{\beta }}_{1}$	−1	20	−0.02	36	−1	26	−0.3	33	0.2	31	−0.1	20
	${\hat{\beta }}_{2}$	−1	20	−2	37	−2	26	−2	33	−1	31	−1	20
III	${\hat{\beta }}_{1}$	−0.03	24	−14	47	1	28	−11	41	0.4	26	1	18
	${\hat{\beta }}_{2}$	−1	25	16	47	−1	29	13	41	−2	26	−0.2	18
	${\hat{\beta }}_{3}$	4	39	−21	78	3	49	−17	68	4	45	2	30
	${\hat{\beta }}_{4}$	0.01	27	22	48	0.3	30	24	43	1	30	1	21
	${\hat{\beta }}_{5}$	0.4	31	104	53	3	38	88	46	1	26	1	18
IV	${\hat{\beta }}_{1}$	0.5	23	1	44	1	27	0.3	38	0.3	24	−0.1	17
	${\hat{\beta }}_{2}$	0.2	23	1	43	1	27	1	37	1	25	1	18
	${\hat{\beta }}_{3}$	0.5	24	5	44	1	28	3	38	1	25	1	17
	${\hat{\beta }}_{4}$	−1	23	−2	45	−1	27	−2	39	−1	25	−1	18
	${\hat{\beta }}_{5}$	2	23	4	44	2	27	3	38	2	24	1	17
		a = 1/5, cen = 50%				a = 1/3, cen = 50%				a = 1, cen = 50%			
		Proposed		MPLE		Proposed		MPLE		Proposed		MPLE	
		Bias	SE	Bias	SE	Bias	SE	Bias	SE	Bias	SE	Bias	SE
I	${\hat{\beta }}_{1}$	1	26	104	70	1	35	94	63	0.02	52	−1	30
	${\hat{\beta }}_{2}$	0.05	26	86	53	−1	35	79	49	−4	53	−3	30
II	${\hat{\beta }}_{1}$	0.4	25	3	46	2	33	2	42	2	38	1	25
	${\hat{\beta }}_{2}$	−0.5	25	−0.3	46	0.05	33	−0.3	41	−0.2	38	0.3	25
III	${\hat{\beta }}_{1}$	−1	35	−10	58	−1	39	−8	51	1	29	1	21
	${\hat{\beta }}_{2}$	−0.1	33	11	58	−1	39	10	51	−2	30	−1	21
	${\hat{\beta }}_{3}$	4	55	−14	99	7	62	−12	85	3	52	1	37
	${\hat{\beta }}_{4}$	−1	37	34	66	−1	40	32	58	−1	38	−1	27
	${\hat{\beta }}_{5}$	5	44	113	64	5	51	95	56	2	29	−0.2	20
IV	${\hat{\beta }}_{1}$	−1	32	2	52	−2	36	1	45	0.2	28	0.1	20
	${\hat{\beta }}_{2}$	−2	32	−3	55	−1	36	−2	48	0.3	30	−0.2	21
	${\hat{\beta }}_{3}$	1	31	3	56	2	36	2	49	1	30	0.3	21
	${\hat{\beta }}_{4}$	−1	31	−4	55	−3	36	−3	47	0.02	28	−0.1	20
	${\hat{\beta }}_{5}$	3	33	6	56	4	37	5	48	4	30	2	21

Note: MPLE stands for the maximum partial likelihood estimator and cen stands for the censoring rate. Bias and SE are the empirical bias (×1000) and empirical standard deviation (×1000) of 1000 simulated datasets, respectively.

**Table 3:** Estimated coefficients for the German Breast Cancer Study.

	Proposed model^1^	Cox model^2^
Hormonal therapy	−0.325 (−0.519, −0.047)	−0.248
Tumor size	0.165 (−0.052, 0.344)	0.073
Tumor grade 2	0.444 (0.124, 0.642)	0.567
Tumor grade 3	0.474 (0.166, 0.843)	0.754
Number of positive nodes	0.667 (0.280, 0.862)	0.207

1 For the proposed model, we reported $-\hat{\beta }$ so that the parameters can be compared with the Cox model. We also reported the 2.5th and 97.5th percentiles from 500 Bootstrap replicates. Note that the coverage probability of this interval may not be close to 95%.

2 For the Cox model, we reported the normalized regression parameters ${\hat{\beta }}_{P}∕\|{\hat{\beta }}_{P}\|$.

## Appendix

### Regularity Conditions

To establish the large sample property of the maximum likelihood estimator, we impose the following conditions:

(C1) Pr(Δ=1)>0 and Pr(Y>τ)>0 for a prespecified constant τ.
(C2) Define $p(Y,\;\Delta \;|\;X;\beta ,\;r,\;\Lambda )={\left\{\lambda (Y)∕r\left(\beta^{T}X\right)\right\}}^{\Delta }\;\text{exp}\{-\Lambda (Y)∕r\left(\beta^{T}X\right)\}$. For any (β,r,Λ)∈(𝓑,𝓡,𝓐), the Kullback-Leilber divergence,
  $$E\;\log\left\{\frac{p(Y,\;\Delta \;|\;X;\;\beta_{0},\;r_{0},\;\Lambda_{0})}{p(Y,\;\Delta \;|\;X;\;\beta ,\;r,\;\Lambda )}\right\},$$
  is strictly larger than zero if ($\left(\beta ,\;r,\;\Lambda )\neq (\beta_{0},\;r_{0},\;\Lambda_{0}\right)$).
(C3) There exit constants 0<a<b<∞ so that $a\leq {\text{inf}}_{z\in R}\;r(z)\leq {\text{sup}}_{z\in R}r(z)\leq b$, ∀r(⋅)∈𝓡.
(C4) The support of X is a bounded convex set of $R^{d}$. For any β∈𝓑, the density of $\beta^{T}X$ is bounded from above and below by some constants.
(C5) The quantity $E\left[\Delta r_{0}^{2}\left(X^{T}\beta_{0}\right){\left\{r^{-1}\left(X^{T}\beta \right)-r_{0}^{-1}\left(X^{T}\beta_{0}\right)\right\}}^{2}\right]-E\Delta {\left\{S(Y,\;r,\;\beta )-S(Y,r_{0},\;\beta_{0})\right\}}^{2}S^{-2}(Y,\;r_{0},\;\beta_{0})$ is bounded from below and above up to some constants by $E{\left\{r\left(X^{T}\beta \right)-r_{0}\left(X^{T}\beta_{0}\right)\right\}}^{2}$, where $S(y,\;r,\;\beta )=E\{I(Y\geq y)∕r(X^{T}\beta )\}$.

### Proof of Model Identifiability

Assume that there exists two sets of parameters {$\beta_{1},\lambda_{1}(t),r_{1}(z))$} and {$\beta_{2},\lambda_{2}(t),r_{2}(z))$}, with $\int_{0}^{\tau }\lambda_{1}(t)dt=\int_{0}^{\tau }\lambda_{2}(t)dt=1$, $r_{1}(z)$ and $r_{2}(z)$ being non-decreasing, and $\|\beta_{1}\|=\|\beta_{2}\|=1$, that give the same conditional hazard function, that is,

$$\frac{\lambda_{1}(t)}{r_{1}(x^{T}\beta_{1})}=\frac{\lambda_{2}(t)}{r_{2}(x^{T}\beta_{2})}\;\;\text{for all}\;t,x,$$

or, equivalently,

$$\frac{\lambda_{1}(t)}{\lambda_{2}(t)}=\frac{r_{1}(x^{T}\beta_{1})}{r_{2}(x^{T}\beta_{2})}\;\;\text{for all}\;t,x.$$

We want to show that $\lambda_{1}=\lambda_{2}$, $r_{1}=r_{2}$, and $\beta_{1}=\beta_{2}$. Since the left-hand-side of the equality depends only on t while the right-hand-side depends only on x, the equality holds if and only if there exists a constant k such that

$$\lambda_{1}(t)=k\;\lambda_{2}(t)\;\;\text{and}\;\;r_{1}(x^{T}\beta_{1})=k\;r_{2}(x^{T}\beta_{2})\;\text{for all}\;t,x.$$

The identifiability condition $\int_{0}^{\tau }\lambda_{1}(t)dt=\int_{0}^{\tau }\lambda_{2}(t)dt=1$ implies k=1, and hence we have $\lambda_{1}(t)=\lambda_{2}(t)$ for all t and $r_{1}(x\beta_{1})=r_{2}(x\beta_{2})$ for all x. It is clear that $r_{1}\equiv r_{2}$ under the monotone constraint if $\beta_{1}=\beta_{2}$, hence it suffices to show $\beta_{1}=\beta_{2}$.

Since r is continuous and nonconstant, there exists $B(x_{0},\kappa )=\{x_{0}+\gamma u,\;\|\gamma \|=1,u\in [-\kappa ,\kappa ]\}$ for some $x_{0}$ such that r is nonconstant on $B(x_{0},\kappa )$. For any w∈[−κ,κ], it follows from the identifiability condition $\|\beta_{1}\|=\beta_{1}^{T}\beta_{1}=1$ that

$$r_{1}(x_{0}^{T}\beta_{1}+u)=r_{1}({\left(x_{0}+u\beta_{1}\right)}^{T}\beta_{1})=r_{2}({\left(x_{0}+u\beta_{1}\right)}^{T}\beta_{2})=r_{2}(x_{0}^{T}\beta_{2}+\beta_{1}^{T}\beta_{2}u),$$

and, similarly,

$$r_{2}(x_{0}^{T}\beta_{2}+u)=r_{2}({\left(x_{0}+u\beta_{2}\right)}^{T}\beta_{2})=r_{1}({\left(x_{0}+u\beta_{2}\right)}^{T}\beta_{1})=r_{1}(x_{0}^{T}\beta_{1}+\beta_{2}^{T}\beta_{1}u).$$

Without loss of generalizability, we assume that the first element of β is positive. So if $\beta_{1}\neq \beta_{2}$, we have $|\beta_{1}^{T}\beta_{2}|<1$ by Cauchy-Schwartz inequality. As a result,

$$r_{1}(x_{0}^{T}\beta_{1}+u)\;=\;r_{2}(x_{0}^{T}\beta_{2}+\beta_{1}^{T}\beta_{2}u)=r_{1}(x_{0}^{T}\beta_{1}+{\left(\beta_{1}^{T}\beta_{2}\right)}^{2}u)=\cdots =r_{1}(x_{0}^{T}\beta_{1}).$$

For any $x\in B(x_{0},\kappa )$, we can express it as $x=x_{0}+\gamma w$ for some unit vector γ and w∈[−κ,κ]. Thus we have $r_{1}(x^{T}\beta_{1})=r_{1}(x_{0}^{T}\beta_{1}+\gamma^{T}\beta_{1}w)=r_{1}(x_{0}^{T}\beta_{1})$. This implies $r_{1}$ is constant on $B(x_{0},\kappa )$, which is a contradiction. Hence we show that $\beta_{1}=\beta_{2}$, and therefore $r_{1}\equiv r_{2}$.

### Proof of Theorem 1

We denote expectation with respect to the empirical distribution of the data by $P_{n}$ and denote expectation with respect to the true underlying distribution of the data by P. Define

$$\ell (\beta ,\;r,\;\Lambda )=-\delta \;\log\;r(x^{T}\beta )+\delta \;\log\;d\;\Lambda (y)-\Lambda (y)∕r(x^{T}\beta ),$$

where dΛ(y)=Λ(y)−Λ(y−). Let ($\hat{\beta }$, $\hat{r}$, $\hat{\Lambda }$) be the maximizer of the likelihood under the constraints, that is, for any (β, r, Λ),

$$P_{n}\ell (\hat{\beta },\;\hat{r},\;\hat{\Lambda })\geq P_{n}\ell (\beta ,\;r,\;\Lambda ),$$

where $\hat{\Lambda }$, Λ are monotonically nondecreasing functions satisfying $\hat{\Lambda }(0)=\Lambda (0)=0$ and $\hat{\Lambda }(\tau )=\Lambda (\tau )=1,\hat{r},r$ are monotonically nondecreasing and bounded functions, and $\|\hat{\beta }\|=\|\beta \|=1$. Define N(t)=ΔI(Y≤t) and R(t)=I(Y≥t). Let

$${\hat{\Lambda }}_{1}(t)=\int_{0}^{t}\frac{\sum_{i=1}^{n}d\;N_{i}(u)}{\sum_{i=1}^{n}R_{i}(u)∕\hat{r}(x_{i}^{T}\hat{\beta })}$$

be the Breslow type estimator. The constrained MLE for Λ is the normalized version

$$\hat{\Lambda }(t)={\hat{\Lambda }}_{1}(t)∕{\hat{\Lambda }}_{1}(\tau ).$$

Since $\hat{r}(\cdot )$ is a bounded monotonic function and has bounded variation, by Helly’s selection theorem, it has a convergence subsequence to a function $r^{*}(\cdot )$. Moreover, since $\hat{\beta }$ falls in a compact subset of $R^{p}$, it also has a convergence subsequence to a limiting value $\beta^{*}$. Then there exists a further subsequence {$n_{k}$} along which $\hat{r}(z)\to r^{*}(z)$ and $\|{\hat{\beta }-\beta }^{*}\|\to 0$, and thus along {$n_{k}$}, $\hat{\Lambda }(t)$ converges almost surely to $\Lambda^{*}(t)$, where $\Lambda^{*}(t)=\Lambda_{1}^{*}(t)∕\Lambda_{1}^{*}(\tau )$ and $\Lambda_{1}^{*}(t)=\int_{0}^{t}\frac{P\{dN(u)\}}{P\{R(u)∕r^{*}(x^{T}\beta^{*})\}}$.

Define

$${\tilde{\Lambda }}_{1}(t)=\int_{0}^{t}\frac{\sum_{i=1}^{n}d\;N_{i}(u)}{\sum_{i=1}^{n}R_{i}(u)∕r_{0}(x_{i}^{T}\beta_{0})},\;\tilde{\Lambda }(t)={\tilde{\Lambda }}_{1}(t)∕{\tilde{\Lambda }}_{1}(\tau ).$$

By Lemma 9.10 and Corollary 9.27 in Kosorok (Kosorok, 2008), it can be seen that {N(t):t≥0}} and {$R(t)∕r_{0}(x^{T}\beta_{0})\;:\;t\geq 0]$} are Glivenko-Cantelli classes. Then $\tilde{\Lambda }(t)$ converges almost surely to $\Lambda_{0}(t)$, where $\Lambda_{1}^{0}(t)=\int_{0}^{t}\frac{P\{dN(u)\}}{P\{R(u)∕r_{0}(x^{T}\beta_{0})\}}$ and $\Lambda_{0}(t)=\Lambda_{1}^{0}(t)∕\Lambda_{1}^{0}(\tau )$.

Note that {$\delta \;\log\;r(x^{T}\beta )-\Lambda (y)∕r(x^{T}\beta )\;:\;(\beta ,\;r,\;\Lambda )\in (𝓑,\;𝓡,\;𝓐)$} is a Glivenko-Cantelli class since it is indexed by monotonic functions Λ, r and parameters β and it has an integrable envelop function (Theorem 3, van der Vaart and Wellner (van der Vaart and Wellner, 2000)). Then we have

$$\;P_{n_{k}}\{\ell (\hat{\beta },\hat{r},\hat{\Lambda })-\ell (\beta_{0},r_{0},\tilde{\Lambda })\}\;=\;-P_{n_{k}}\delta \;\log\;\frac{\hat{r}(x^{T}\hat{\beta })}{r_{0}(x^{T}\beta_{0})}+P_{n_{k}}\delta \;\log\;\frac{d\hat{\Lambda }(y)}{d\tilde{\Lambda }(y)}-P_{n_{k}}\{\hat{\Lambda }(y)∕\hat{r}(x^{T}\hat{\beta })-\tilde{\Lambda }(y)∕r_{0}(x^{T}\beta_{0})\}\;=\;-P_{n_{k}}\delta \;\log\;\frac{\hat{r}(x^{T}\hat{\beta })}{r_{0}(x^{T}\beta_{0})}+P_{n_{k}}\delta \;\log\;\frac{{\tilde{\Lambda }}_{1}(\tau )P_{n_{k}}\{R(y)∕r_{0}(x^{T}\beta_{0})\}}{{\hat{\Lambda }}_{1}(\tau )P_{n_{k}}\{R(y)∕\hat{r}(x^{T}\hat{\beta })\}}-P_{n_{k}}\{\hat{\Lambda }(y)∕\hat{r}(x^{T}\hat{\beta })-\tilde{\Lambda }(y)∕r_{0}(x^{T}\beta_{0})\}\;\overset{a.s.}{\to }\;-P\delta \;\log\;\frac{r^{*}(x^{T}\beta^{*})}{r_{0}(x^{T}\beta_{0})}+P\delta \;\log\;\frac{\Lambda_{1}^{0}(\tau )P\{R(y)∕r_{0}(x^{T}\beta_{0})\}}{\Lambda_{1}^{*}(\tau )P\{R(y)∕r^{*}(x^{T}\beta^{*})\}}-P\{\Lambda^{*}(y)∕r^{*}(x^{T}\beta^{*})-\Lambda_{0}(y)∕r_{0}(x^{T}\beta_{0})\}\;=\;-P\delta \;\log\;\frac{r^{*}(x^{T}\beta^{*})}{r_{0}(x^{T}\beta_{0})}+P\delta \;\log\;\frac{d\Lambda^{*}(y)}{d\Lambda_{0}(y)}-P\{\Lambda^{*}(y)∕r^{*}(x^{T}\beta^{*})-\Lambda_{0}(y)∕r_{0}(x^{T}\beta_{0})\}.$$

Therefore, we obtain $0\leq P_{n_{k}}\{\ell (\hat{\beta },\hat{r},\hat{\Lambda })-\ell (\beta_{0},r_{0},\tilde{\Lambda })\}\to P\;\log\;\frac{dP_{\beta^{*},r^{*},\Lambda^{*}}}{dP_{\beta_{0},r_{0},\Lambda_{0}}}$, where $P_{\beta ,r,\Lambda }$ is the probability measure of a single observation on the specified model at parameter value (β,r,Λ). By identifiability of the model, we have

$$\Lambda^{*}=\Lambda_{0},r^{*}=r_{0},\beta^{*}=\beta_{0}.$$

Therefore, we have shown that any convergence subsequence has a limiting to the true underlying parameters. Since every subsequence of n contains a further subsequence for which ($\hat{\beta },\hat{r},\hat{\Lambda }$) converges uniformly to ($\beta_{0}$, $r_{0}$, $\Lambda_{0}$), we have convergence for the entire sequence.

### Proof of Proposition 1

We need to show that there exists a constant $c^{*}<0$ such that $\beta =c^{*}\beta_{0}$ is the solution to the limiting value of the partial score equation,

(2)
$$U(\beta )=E\{XN(\tau )\}-\int_{0}^{\tau }\frac{E\{X\;\text{exp}(X^{T}\beta )I(Y\geq t)\}}{E\{\text{exp}(X^{T}\beta )I(Y\geq t)\}}E\{dN(t)\}=0,$$

where N(t)=ΔI(Y≤t). Without loss of generality, we assume E(X)=0. Define $W=X^{T}\beta_{0}$. Using the property of elliptically symmetric random variables, we have $E\{X\;\;W\}=W_{b}$, where b is a p×1 deterministic vector such that $b=\Sigma \beta_{0}{\left(\beta_{0}^{T}\Sigma \beta_{0}\right)}^{-1}$ and Σ=var(X). Under random censoring, it can be shown that for any c∈R,

$$E\{X\;\text{exp}({cX}^{T}\beta_{0})I(Y\geq t)\}=E\{\text{exp}(cW)I(Y\geq t)W\}b,\;\;E\{\text{exp}({cX}^{T}\beta_{0})I(Y\geq t)\}=E\{\text{exp}(cW)I(Y\geq t)\},\;\;E\{XdN(u)\}=E\{WdN(u)\}b.$$

By plugging the above quantities into (2), we have

$$U(c\beta_{0})=b\left[E\{WN(\tau )\}-\int_{0}^{\tau }\frac{E\{W\;\text{exp}(cW)I(Y\geq t)\}}{E\{\text{exp}(cW)I(Y\geq t)\}}E\{dN(t)\}\right].$$

Le $c^{*}$ be the limiting value of estimated c as n→∞ from fitting the Cox model λ(t∣W)=λ(t)exp{cW} under the true model λ(t∣W)=λ(t)exp{−ϕ(W)}. Then we have $U(c^{*}\beta_{0})=0$. Moreover, the monotonicity of ϕ will result in a negative value of $c^{*}$.
