# Supplementary material for: Maximum Likelihood Estimation for Shape-restricted Single-index Hazard Models
Source: J Data Sci. Author manuscript; Available in PMC 2024 Apr 15. (PMC11017303; doi:10.6339/22-jds1061)
Supplement: supplemental materials [file NIHMS1933356-supplement-supplemental_materials.pdf]

Supplementary Information for "Maximum likelihood estimation for shape-restricted single-index hazard models"

Jing Qin* Yifei Sun Ao Yuan Chiung-Yu Huang

1 Proof for Theorem 2

We first show $[P_X\{\widehat{r}(X^\intercal\widehat{\beta}) - r_0(X^\intercal\beta_0)\}^2]^{1/2} = O_p(n^{-1/3})$ by applying Theorem 3.4.1 in van der Vaart and Weller [1996]. Define

$$\widehat{S}(y, r, \beta) = P_n\{I(Y \geq y)/r(X^\intercal\beta)\},$$

and

$$\begin{aligned} S(y, r, \beta) &= P\{I(Y \geq y)/r(X^\intercal\beta)\} \\ &= \int_0^\infty \bar{G}(y \mid x)\exp\{-\Lambda_0(y)/r_0(x^\intercal\beta_0)\}r^{-1}(x^\intercal\beta)dP_X(x), \end{aligned} \tag{1}$$

where $\bar{G}(y \mid x)$ is the conditional survival function of the censoring time C. The partial log-likelihood can then be written as

$$M_n(r, \beta) = P_n\Delta[\log r^{-1}(X^\intercal\beta) - \log\widehat{S}(Y, r, \beta)].$$

We also define its limiting function,

$$M(r, \beta) = P\Delta[\log r^{-1}(X^\intercal\beta) - \log S(Y, r, \beta)].$$

In what follows, the notation $a \lesssim b$ ($a \gtrsim b$) means that a is bounded above (below) by b up to a universal positive constant. Define $d(r, r_0; \beta, \beta_0) = [P\{r(X^\intercal\beta) - r_0(X^\intercal\beta_0)\}^2]^{1/2}$ for $\beta \in \mathcal{B}$ and $r \in \mathcal{R}$. We need to show that, for any $\delta > 0$,

$$\sup_{\delta/2 \leq d(r, r_0; \beta, \beta_0) \leq \delta, (\beta, r) \in (\mathcal{B}, \mathcal{R})} M(r, \beta) - M(r_0, \beta_0) \lesssim -\delta^2.$$

In what follows, we will show

$$M(r, \beta) - M(r_0, \beta_0) \lesssim -d(r, r_0; \beta, \beta_0)^2.$$

Consider the Taylor expansion

$$\log(x) - \log(x_0) = \frac{x - x_0}{x_0} - \frac{1}{2x_0^2}(x - x_0)^2 + \frac{1}{3\xi^3}(x - x_0)^3,$$

where ξ lies between x_0 and x. Then we have

$$\begin{aligned} &M(r, \beta) - M(r_0, \beta_0) \\ &= P\Delta\left[r_0(X^\intercal\beta_0)\{r^{-1}(X^\intercal\beta) - r_0^{-1}(X^\intercal\beta_0)\} - \frac{S(Y, r, \beta) - S(Y, r_0, \beta_0)}{S(Y, r_0, \beta_0)}\right] \\ &\quad - P\Delta\left[\frac{1}{2}r_0^2(X^\intercal\beta_0)\{r^{-1}(X^\intercal\beta) - r_0^{-1}(X^\intercal\beta_0)\}^2 - \frac{\{S(Y, r, \beta) - S(Y, r_0, \beta_0)\}^2}{2S^2(Y, r_0, \beta_0)}\right] \\ &\quad + P\frac{\Delta}{3}\left[\tilde{r}^3(X^\intercal\tilde{\beta})\{r^{-1}(X^\intercal\beta) - r_0^{-1}(X^\intercal\beta_0)\}^3 - \frac{\{S(Y, r, \beta) - S(Y, r_0, \beta_0)\}^3}{S^3(Y, \tilde{r}, \tilde{\beta})}\right] \\ &:= I + II + III. \end{aligned}$$

*Corresponding author. Email: jingqin@niaid.nih.gov

First, we will show that $I = 0$. Let $f(t \mid x)$ and $S(t \mid x)$ be the conditional density and survival functions of T given $X = x$. Then $f(t \mid x) = \lambda_0(t)/r_0(x^\intercal\beta_0)\exp\{-\Lambda_0(t)/r_0(x^\intercal\beta_0)\}$, and

$$\begin{aligned} E(\Delta \mid X = x) &= \int_0^\infty P(C \geq t \mid x) f(t \mid x) dt \\ &= \int_0^\infty \bar{G}(t \mid x) r_0^{-1}(x^\intercal\beta_0)\exp\{-\Lambda_0(t)/r_0(x^\intercal\beta_0)\} d\Lambda_0(t). \end{aligned}$$

For any function H,

$$\begin{aligned} &P\Delta H(Y)/S(Y, r_0, \beta_0) \qquad (2)\\ =& \int_0^\infty P_X\{\bar{G}(y|X) r_0^{-1}(X^\intercal\beta_0)\exp(-\Lambda_0(y)/r_0(X^\intercal\beta_0))\} H(y) S^{-1}(y, r_0, \beta_0) d\Lambda_0(y) \\ =& \int_0^\infty S(y, r_0, \beta_0) H(y) S^{-1}(y, r_0, \beta_0) d\Lambda_0(y) \quad \text{(by (1))} \\ =& \int_0^\infty H(y) d\Lambda_0(y). \end{aligned}$$

Thus, we have

$$\begin{aligned} &P\Delta r_0(X^\intercal\beta_0)\{r^{-1}(X^\intercal\beta) - r_0^{-1}(X^\intercal\beta_0)\} \\ =&P\int_0^\infty \bar{G}(y \mid X)\exp\{-\Lambda_0(y)/r_0(X^\intercal\beta_0)\}\{r^{-1}(X^\intercal\beta) - r_0^{-1}(X^\intercal\beta_0)\} d\Lambda_0(y) \\ =&\int_0^\infty \{S(y, r, \beta) - S(y, r_0, \beta_0)\} d\Lambda_0(y) \\ =&P\Delta \frac{S(Y, r, \beta) - S(Y, r_0, \beta_0)}{S(Y, r_0, \beta_0)} \quad \text{(by (2))}. \end{aligned}$$

Therefore, we have proved $I = 0$.

Second, we consider II. Define

$$\begin{aligned} A(r, \beta) &= P\big[\Delta r_0^2(X^\intercal\beta_0)\{r^{-1}(X^\intercal\beta) - r_0^{-1}(X^\intercal\beta_0)\}^2\big], \\ B(r, \beta) &= P\Delta\{S(Y, r, \beta) - S(Y, r_0, \beta_0)\}^2 S^{-2}(Y, r_0, \beta_0). \end{aligned}$$

Then we have $II = -0.5\{A(r, \beta) - B(r, \beta)\}$. By Cauchy–Schwarz inequality,

$$\begin{aligned} B(r, \beta) =& \int_0^\infty S^{-1}(y, r_0, \beta_0)[P_X \bar{G}^{1/2}(y|X) r_0^{-1/2}(X^\intercal\beta)\exp\{-0.5\Lambda_0(y)/r_0(X^\intercal\beta_0)\}\bar{G}^{1/2}(y|X) \\ &\times r_0^{1/2}(X^\intercal\beta_0)\big(r^{-1}(X^\intercal\beta) - r_0^{-1}(X^\intercal\beta_0)\big)\exp\{-0.5\Lambda_0(y)/r_0(X^\intercal\beta_0)\}]^2 d\Lambda_0(y) \\ \leq& \int_0^\infty S^{-1}(y, r_0, \beta_0) P_X\{\bar{G}(y|X) r_0^{-1}(X^\intercal\beta_0)\exp\{-\Lambda_0(y)/r_0(X^\intercal\beta_0)\} P_X\{\bar{G}(y|X) \\ &\times r_0(X^\intercal\beta_0)\big(r^{-1}(X^\intercal\beta) - r_0^{-1}(X^\intercal\beta_0)\big)^2\exp\{-\Lambda_0(y)/r_0(X^\intercal\beta_0)\}\} d\Lambda_0(y) \\ =& \int_0^\infty P_X\{\bar{G}(y|X) r_0(X^\intercal\beta_0)\big(r^{-1}(X^\intercal\beta) - r_0^{-1}(X^\intercal\beta_0)\big)^2\exp\{-\Lambda_0(y)/r_0(X^\intercal\beta_0)\} d\Lambda_0(y) \\ =& A(r, \beta). \end{aligned}$$

Moreover, under (C5), we can bound $A(r, \beta) - B(r, \beta)$ from below such that

$$A(r, \beta) - B(r, \beta) \gtrsim P_X\{r(X^\intercal\beta) - r_0(X^\intercal\beta_0)\}^2.$$

Therefore, we have shown $II \lesssim -d(r, r_0; \beta, \beta_0)^2$.

For III, we show III is of a smaller order than $d(r, r_0; \beta, \beta_0)^2$. For some $(\tilde{r}, \tilde{\beta})$, the third term is

$$P\frac{\Delta}{3}\left[\tilde{r}^3(X^\intercal\tilde{\beta})\{r^{-1}(X^\intercal\beta) - r_0^{-1}(X^\intercal\beta_0)\}^3\right] - P\frac{\Delta}{3}\left[\frac{\{S(Y, r, \beta) - S(Y, r_0, \beta_0)\}^3}{S^3(Y, \tilde{r}, \tilde{\beta})}\right].$$

By (C3), we have

$$\left|P\Delta\left[\tilde{r}^3(X^\intercal\tilde{\beta})\{r^{-1}(X^\intercal\beta)-r_0^{-1}(X^\intercal\beta_0)\}^3\right]\right|\lesssim d(r,r_0,\beta,\beta_0)A(r,\beta),$$

$$\left|P\Delta\left[\frac{\{S(Y,r,\beta)-S(Y,r_0,\beta_0)\}^3}{S^3(Y,\tilde{r},\tilde{\beta})}\right]\right|\lesssim d(r,r_0;\beta,\beta_0)B(r,\beta).$$

Since $A(r,\beta)-B(r,\beta)$ is bounded from above up to some constant by $d(r,r_0;\beta,\beta_0)^2$. the third term in the Taylor expansion is a smaller order of $d(r,r_0;\beta,\beta_0)^2$.

As a consequence, we have

$$M(r,\beta)-M(r_0,\beta_0)\lesssim -d(r,r_0;\beta,\beta_0)^2.$$

Denote by E^* the expectation with respect to outer measure. Define $[a]^+=a$ if $a>0$ and $[a]^+=0$ otherwise. Next we show that, for any small $\delta>0$,

$$E^*\sup_{d(r,r_0;\beta,\beta_0)\le\delta,(\beta,r)\in(\mathcal{B},\mathcal{R})}\left[\{M_n(r,\beta)-M(r,\beta)\}-\{M_n(r_0,\beta_0)-M(r_0,\beta_0)\}\right]^+\lesssim\frac{\phi_n(\delta)}{\sqrt{n}},\tag{3}$$

where $\phi_n(\delta)=\delta^{1/2}(1+\delta^{-3/2}n^{-1/2})$. Note that

$$\begin{aligned}&M_n(r,\beta)-M(r,\beta)\\&=(P_n-P)\Delta\log r(X^\intercal\beta)+P_n\Delta\log\widehat{S}(Y,r,\beta)-P\Delta\log S(Y,r,\beta)\\&=(P_n-P)\Delta\log r(X^\intercal\beta)+(P_n-P)\Delta\log\widehat{S}(Y,r,\beta)+P\Delta\log\{\widehat{S}(Y,r,\beta)/S(Y,r,\beta)\}.\end{aligned}$$

Therefore, we have

$$\begin{aligned}&\{M_n(r,\beta)-M(r,\beta)\}-\{M_n(r_0,\beta_0)-M(r_0,\beta_0)\}\\&=[(P_n-P)\Delta\{\log r(X^\intercal\beta)-\log r_0(X^\intercal\beta_0)\}]+[(P_n-P)\Delta\{\log\widehat{S}(Y,r,\beta)-\log\widehat{S}(Y,r_0,\beta_0)\}]\\&\quad+[P\Delta\log\{\widehat{S}(Y,r,\beta)/S(Y,r,\beta)\}-P\Delta\log\{\widehat{S}(Y,r_0,\beta_0)/S(Y,r_0,\beta_0)\}]\\&=[(P_n-P)\Delta\{\log r(X^\intercal\beta)-\log r_0(X^\intercal\beta_0)\}]+[(P_n-P)\Delta\{\log S(Y,r,\beta)-\log S(Y,r_0,\beta_0)\}]\\&\quad+o_p(n^{-1/2})+O_p(\delta n^{-1/2}).\end{aligned}$$

Following Balabdaoui et al. [2019], the ϵ bracketing entropy with the $L_2(P)$ norm for $\{\Delta\{\log r(X^\intercal\beta)-\log r_0(X^\intercal\beta_0)\}:(\beta,r)\in(\mathcal{B},\mathcal{R})\}$ and $\{\Delta\{\log S(Y,r,\beta)-\log S(Y,r_0,\beta_0)\}:(\beta,r)\in(\mathcal{B},\mathcal{R})\}$ is bounded by c/ϵ for some positive constant c.

Recall $M(r,\beta)=P\Delta[\log r^{-1}(X^\intercal\beta)-\log S(Y,r,\beta)]:=Pm(Y,X|r,\beta)$, then $M_n(r,\beta)=P_nm(Y,X|r,\beta)$. Let $q(Y,X|r,\beta)=m(Y,X|r,\beta)-m(Y,X|r_0,\beta_0)$, and $\mathbb{G}_nq=\sqrt{n}(P_n-P)q$. Then we have

$$\sqrt{n}\big(\{M_n(r,\beta)-M(r,\beta)\}-\{M_n(r,\beta)-M(r,\beta)\}\big)=\mathbb{G}_nq(Y,X|r,\beta).$$

Define $\mathcal{Q}(\delta)=\{q(Y,X|r,\beta):(\beta,r)\in(\mathcal{B},\mathcal{R}),d(\beta,\beta_0;r,r_0)\le\delta\}$ and $\|\mathbb{G}_n\|_{\mathcal{Q}(\delta)}=\sup_{q\in\mathcal{Q}(\delta)}|\mathbb{G}_nq|$. The condition $d(\beta,\beta_0;r,r_0)\le\delta$ and continuity of q in (r,β) implies $Pq^2\lesssim\delta^2$ for all $q\in\mathcal{Q}(\delta)$, and the given conditions imply $\sup_{y,x}|q(y,x|r,\beta)|<\bar{q}$ for some constant $0<\bar{q}<\infty$. Let $N_{[]}(\epsilon,\mathcal{Q}(\delta),L_2(P))$ be the ϵ-bracketing number of $\mathcal{Q}(\delta)$. We then have $\log N_{[]}(\epsilon,\mathcal{Q}(\delta),L_2(P))\lesssim 1/\epsilon$, and

$$J_{[]}(\delta,\mathcal{Q}(\delta),L_2(P)):=\int_0^\delta\sqrt{1+\log N_{[]}(\epsilon,\mathcal{Q}(\delta),L_2(P))}d\epsilon\lesssim\delta^{1/2}.$$

By Lemma 3.4.2 of van der Vaart and Weller [1996], the left hand side of (3) is bounded above up to a constant by

$$J_{[]}(\delta,\mathcal{Q}(\delta),L_2(P))\left\{1+\frac{J_{[]}(\delta,\mathcal{Q}(\delta),L_2(P))}{\delta^2\sqrt{n}}\bar{q}\right\}/\sqrt{n}\lesssim\delta^{1/2}(1+\delta^{-3/2}n^{-1/2})/\sqrt{n},$$

which implies (3) with $\phi_n(\delta) = \delta^{1/2}(1 + \delta^{-3/2}n^{-1/2})$. Take $r_n = n^{1/3}$, then

$$r_n^2 \phi_n\Big(\frac{1}{r_n}\Big) \lesssim \sqrt{n}.$$

Collectively, conditions of Theorem 3.4.1 in van der Vaart and Weller [1996] are satisfied. Thus we establish $r_n d(\widehat{\beta}_n, \widehat{r}_n; \beta_0, r_0) = n^{1/3} d(\widehat{\beta}_n, \widehat{r}_n; \beta_0, r_0) = O_p(1)$. Following the arguments in Corollary 5.3 in Balabdaoui et al. [2019], we have $\|\widehat{\beta} - \beta_0\| + \|\widehat{r} - r_0\| = O_p(n^{-1/3})$. Since $\widehat{\Lambda}(\cdot)$ is the Breslow-type estimator, with (C3) the above also implies $\|\widehat{\Lambda} - \Lambda_0\| = O_p(n^{-1/3})$, which completes the proof.

2 Additional simulation results

For comparison, we consider estimating the unknown link function via kernel smoothing in simulations. Specifically, instead of estimating the link function $r(\cdot)$ using PAVA, we derive the kernel-smoothed estimator

$$\widetilde{r}(z; \beta, \Lambda) = \frac{\sum_{i=1}^n K_h(X_i^\intercal \beta - z)\Lambda(Y_i)}{\sum_{i=1}^n K_h(X_i^\intercal \beta - z)\Delta_i},$$

where $K(\cdot)$ is a symmetric kernel function with a bounded support on $[-1, 1]$, h is the bandwidth parameter, and $K_h(\cdot) = K(\cdot/h)/h$ is the scaled kernel function. It can be shown that $\widetilde{r}(z; \beta_0, \Lambda_0)$ consistently estimates $r(z)$ under some regularity conditions.

The estimation procedure is described below. For a given β, we alternate between (M1') and (M2') below repeatedly until the convergence criteria are met. Specifically, suppose the parameter values in the bth step given by $(\Lambda^{(b)}, r^{(b)})$. Then in the $(b+1)$th step, we update the parameter values as following:

(M1') For $j = 1, \ldots, n$, calculate $r(z_j)$ using

$$r^{(b+1)}(z_j) = \frac{\sum_{i=1}^n K_h(X_i^\intercal \beta - z_j)\Lambda^{(b)}(Y_i)}{\sum_{i=1}^n K_h(X_i^\intercal \beta - z_j)\Delta_i}.$$

(M2') Update Λ with the Breslow-Type estimator

$$\Lambda^{(b+1)*}(t) = \sum_{i=1}^n \frac{\Delta_i I(y_i \le t)}{\sum_{j=1}^n I(y_j \ge y_i)/r^{(b+1)}(z_j)}$$

to obtain $\Lambda^{(b+1)}(t) = \Lambda^{(b+1)*}(t)/\Lambda^{(b+1)*}(\tau)$.

Denote the limit by $(\widetilde{\Lambda}(\cdot; \beta), \widetilde{r}(\cdot; \beta))$. Finally, we plug in $\widetilde{\Lambda}(\cdot; \beta)$ and $\widetilde{r}(\cdot; \beta)$ back to the full likelihood function $\mathcal{L}(\beta, r, \Lambda)$ and estimate β with the maximizer of the function $\mathcal{L}(\beta, \widetilde{r}(\cdot; \beta), \widetilde{\Lambda}(\cdot; \beta))$.

We conducted simulations to compare the kernel smoothing estimator and the proposed MLE under the same simulation settings. The simulation results are summarized in Tables 1 and 2. To ensure identifiability, we assume the first element of the regression parameters estimated by the kernel smoothing approach is negative. Both estimators show improved performance in terms of bias and variance when the sample size increases. With moderate sample sizes, no estimator outperforms another in all scenarios. The proposed MLE generally has smaller variances in scenarios where the link function is not exponential, while the kernel smoothing approach has smaller variances under the Cox model with the exponential link.

Table 1: Summary of simulation studies ($n = 200$)

		a = 1/5, cen = 25%				a = 1/3, cen = 25%				a = 1, cen = 25%			
		Proposed		Kernel		Proposed		Kernel		Proposed		Kernel	
		Bias	SE	Bias	SE	Bias	SE	Bias	SE	Bias	SE	Bias	SE
I	$\widehat{\beta}_1$	2	60	4	69	2	69	3	71	2	88	-2	78
	$\widehat{\beta}_2$	-3	61	-3	70	-5	71	-4	73	-9	89	-12	81
II	$\widehat{\beta}_1$	3	53	2	57	2	61	3	61	4	63	3	61
	$\widehat{\beta}_2$	-1	53	-2	56	-3	62	-2	60	-2	63	-2	61
III	$\widehat{\beta}_1$	6	68	-4	68	4	71	-4	67	-3	52	-4	46
	$\widehat{\beta}_2$	-6	71	-0	72	-5	73	-1	70	-5	52	-3	48
	$\widehat{\beta}_3$	17	119	36	109	16	119	34	105	11	90	21	78
	$\widehat{\beta}_4$	-7	76	3	76	-10	77	4	73	-6	61	1	54
	$\widehat{\beta}_5$	8	94	12	91	10	92	12	87	4	54	-1	49
IV	$\widehat{\beta}_1$	-1	70	1	71	1	71	2	67	0.3	50	1	47
	$\widehat{\beta}_2$	-10	70	-12	67	-13	70	-13	64	-7	50	-6	46
	$\widehat{\beta}_3$	4	67	-2	68	2	68	-3	65	-1	49	-3	46
	$\widehat{\beta}_4$	-7	68	-5	67	-5	70	-4	64	-2	50	-1	46
	$\widehat{\beta}_5$	7	69	10	72	8	71	9	69	6	51	7	46
		a = 1/5, cen = 50%				a = 1/3, cen = 50%				a = 1, cen = 50%			
		Proposed		Kernel		Proposed		Kernel		Proposed		Kernel	
		Bias	SE	Bias	SE	Bias	SE	Bias	SE	Bias	SE	Bias	SE
I	$\widehat{\beta}_1$	3	84	3	87	5	97	3	93	5	107	-7	98
	$\widehat{\beta}_2$	-7	87	-8	89	-8	97	-9	95	-11	111	-22	105
II	$\widehat{\beta}_1$	-0	75	2	72	4	83	1	77	3	77	1	78
	$\widehat{\beta}_2$	-8	76	-5	76	-6	83	-8	82	-6	77	-8	80
III	$\widehat{\beta}_1$	-2	100	-16	100	-1	98	-12	96	1	63	-3	60
	$\widehat{\beta}_2$	-13	100	-7	93	-13	96	-7	91	-5	64	-7	60
	$\widehat{\beta}_3$	25	152	46	143	27	153	45	137	8	106	17	92
	$\widehat{\beta}_4$	-15	107	-0.05	103	-15	105	0.5	100	-12	75	-4	69
	$\widehat{\beta}_5$	30	127	40	122	22	120	29	111	6	60	1	55
IV	$\widehat{\beta}_1$	5	94	1	92	4	93	5	91	0.3	60	-0.2	54
	$\widehat{\beta}_2$	-9	90	-16	98	-8	91	-14	92	-6	62	-7	59
	$\widehat{\beta}_3$	4	95	4	92	6	91	3	87	0.2	62	-2	57
	$\widehat{\beta}_4$	-19	91	-14	91	-19	88	-10	83	-7	58	-2	57
	$\widehat{\beta}_5$	11	93	14	91	9	90	13	89	8	63	11	57

Note: Bias and SE are the empirical bias ($\times 1000$) and empirical standard deviation ($\times 1000$) of 1000 simulated datasets, respectively.

Table 2: Summary of simulation studies ($n = 800$)

		a = 1/5, cen = 25%				a = 1/3, cen = 25%				a = 1, cen = 25%			
		Proposed		Kernel		Proposed		Kernel		Proposed		Kernel	
		Bias	SE	Bias	SE	Bias	SE	Bias	SE	Bias	SE	Bias	SE
I	$\widehat{\beta}_1$	0.1	19	3	26	1	26	2	30	1	44	-1	35
	$\widehat{\beta}_2$	-0.4	19	2	26	-0.1	26	1	30	-2	44	-2	35
II	$\widehat{\beta}_1$	-1	20	-1	23	-1	26	-1	26	0.2	31	0.3	26
	$\widehat{\beta}_2$	-1	20	-1	23	-2	26	-2	26	-1	31	-1	26
III	$\widehat{\beta}_1$	-0.03	24	-1	29	1	28	-1	29	0.4	26	0.3	20
	$\widehat{\beta}_2$	-1	25	1	27	-1	29	1	29	-2	26	1	20
	$\widehat{\beta}_3$	4	39	11	44	3	49	10	47	4	45	8	35
	$\widehat{\beta}_4$	0.01	27	5	32	0.3	30	4	32	1	30	2	24
	$\widehat{\beta}_5$	0.4	31	3	37	3	38	3	38	1	26	-2	20
IV	$\widehat{\beta}_1$	0.5	23	1	26	1	27	1	27	0.3	24	0.1	19
	$\widehat{\beta}_2$	0.2	23	-0.4	27	1	27	-0.04	28	1	25	1	20
	$\widehat{\beta}_3$	0.5	24	0.1	26	1	28	1	28	1	25	1	20
	$\widehat{\beta}_4$	-1	23	-0.1	27	-1	27	0.2	28	-1	25	-1	20
	$\widehat{\beta}_5$	2	23	2	26	2	27	3	27	2	24	1	20
		a = 1/5, cen = 50%				a = 1/3, cen = 50%				a = 1, cen = 50%			
		Proposed		Kernel		Proposed		Kernel		Proposed		Kernel	
		Bias	SE	Bias	SE	Bias	SE	Bias	SE	Bias	SE	Bias	SE
I	$\widehat{\beta}_1$	1	26	1	34	1	35	2	39	0.02	52	-3	45
	$\widehat{\beta}_2$	0.05	26	-1	34	-1	35	-0.2	39	-4	53	-6	46
II	$\widehat{\beta}_1$	0.4	25	2	28	2	33	2	34	2	38	2	33
	$\widehat{\beta}_2$	-0.4	25	1	28	0.05	33	0.4	34	-0.2	38	0.5	33
III	$\widehat{\beta}_1$	-1	35	-4	40	-1	39	-2	42	1	29	-0.3	24
	$\widehat{\beta}_2$	-0.07	33	0.3	37	-1	39	-1	39	-2	30	1	24
	$\widehat{\beta}_3$	4	55	11	58	7	62	10	62	3	52	8	43
	$\widehat{\beta}_4$	-1	37	6	41	-1	40	7	45	-1	38	-1	31
	$\widehat{\beta}_5$	5	44	12	52	5	51	11	51	2	29	-4	23
IV	$\widehat{\beta}_1$	-1	32	0.3	33	-2	36	-0.5	36	0.2	28	0.3	23
	$\widehat{\beta}_2$	-2	32	-1	34	-1	36	-1	36	0.3	30	-0.5	24
	$\widehat{\beta}_3$	1	31	0.4	34	2	36	0.1	36	1	30	1	25
	$\widehat{\beta}_4$	-1	31	-0.3	35	-3	36	-1	37	0.02	28	-0.04	23
	$\widehat{\beta}_5$	3	33	5	37	4	37	5	38	4	30	1	24

Note: Bias and SE are the empirical bias ($\times 1000$) and empirical standard deviation ($\times 1000$) of 1000 simulated datasets, respectively.

References

Fadoua Balabdaoui, Cécile Durot, and Hanna Jankowski. Least squares estimation in the monotone single index model. *Bernoulli*, 25(4B):3276–3310, 2019.

Aad W van der Vaart and Jon Weller. *Weak Convergence and Empirical Processes.* Springer-Verlag, New York, 1996.
